# Study on Strategic Interaction between Government and Farmers in Rural Passive Energy Transformation

**DOI:** 10.3390/ijerph192214862

**Published:** 2022-11-11

**Authors:** Shengyue Fan, Shuai Zha, Chenxi Zhao

**Affiliations:** School of Economics, Minzu University of China, Beijing 100081, China

**Keywords:** rural passive energy transformation, government policy characteristics, farmers’ behavior intentions, interactive study

## Abstract

In the implementation of public environmental policies, the deviation between the behavioral intention of farmers and the results of policy implementation is widespread. To reveal the universality, and break through existing research perspectives, this paper, starting from the interaction between the government and farmers, builds a rural passive energy transformation mechanism conceptual model. Using the policy of “coal to gas” in northern China area as a case, a comprehensive analysis of the influencing factors of peasant household behavior response is presented, using a structural equation to compute the interaction strength between the two agents. The results of the study show that: (1) the standardized path coefficients of household behavioral intention and government policy characteristics on household behavioral response are 0.458 and 0.554, respectively. The effect of government is stronger than that of households, which highlights the change effect of government on household behavioral responses and explains the reason for the deviation between behavioral intention and behavioral response; and (2) The standardized correlation coefficient between government policy characteristics and farmers’ behavioral intention is 0.858, indicating that the interaction between government and farmers has a significant impact on policy results, and verifies the important role of research on the interactions between government and farmers. Therefore, in order to improve the effect of rural energy transformation, it is necessary to strengthen the interactions between the government and farmers, to smooth the channels of farmers’ demands, and to form a pattern of air pollution control with government guidance and full participation.

## 1. Introduction

China is the world’s largest producer and consumer of coal, and coal combustion is widely recognized as a major source of ambient air pollution in China, where the main causes of pollutant emissions are energy intensity and energy structure [1]. Households are the end consumption sector of energy, and their energy consumption behavior influences 45–55% of all energy consumption [2]. According to statistical data, the average annual household energy consumption of rural residents increased from 155 kg in 2005 to 417 kg of standard coal in 2017 [3], which means that rural households consume a huge amount of energy due to the increase in the average annual household energy consumption of rural residents [4,5,6]. Therefore, the implementation of energy transition by replacing coal with clean energy sources such as natural gas is considered an important means to combat air pollution in China [7]. In 2017, the Chinese government introduced an air pollution control program that required the “2 + 26” cities to complete the replacement of coal with electricity and gas in more than 3 million households in that year [8]. The ultimate goal is to achieve improvement of air quality in Beijing, Tianjin, Hebei, and surrounding areas, and to optimize the energy structure in rural areas [9].

According to the energy ladder theory, with the development of the social economy, individual energy consumers will independently change from low-end energy consumption to high-end energy consumption [10]. The energy transformation policy implemented at this stage has broken the order of energy upgrading by families, making low-income farmers represent the status of passive energy transformation. Therefore, the government’s energy subsidies have become the main force to promote the energy transformation in rural areas. As a result, in the current research on energy transition issues, scholars have mostly focused on the study of the impact of government energy subsidies on the energy consumption behavior of farms, mainly analyzing the effect that changes in energy subsidies will affect the implementation of energy transition policies [11,12,13,14]. 

However, the theory of “Decision Reduction and Implementation consultation” of public policies in China holds that [15,16,17] the process of policy implementation is a process of mutual negotiation and bargaining between local governments and the public. As a public policy, the local government not only plays a supervisory role in the process of policy implementation, but also participates as an actor. Therefore, the local government acts as the implementer of the policy and the farmer acts as the recipient of the policy, both of which function together [18]. At present, from the perspective of the two actors, the existing literature considers the interaction mode and intensity of the two actors and neglects the interaction between the government and the farmers, which leads to many difficulties in the process of policy implementation, such as tight natural gas supply, inadequate security and after-sales service, hasty policy implementation, farmers’ behavioral intentions and behavioral response deviations [11,19].

As a representative of authoritarian environmentalism in China, the intensity of the government’s role in the large-scale implementation of energy transition and the interaction between the government and farmers is a topic of great interest and is currently less studied. This paper focuses on the actors involved in the implementation of energy transition policy and analyzes the strength of the interaction between farmers and the government. This model provides a complete picture of the whole process of energy transition policy implementation, thus providing guidance for improving the energy transition policy.

Starting from the analysis framework of the theory of planned behavior, this paper establishes the behavioral intentions of farmers and the government and their driving factors, constructs a model of the joint role of farmers and the government, and forms a theoretical analysis framework of the results of energy transition policy implementation, which provides a new perspective for policy analysis. It also explains the reasons for the deviations between farmers’ behavioral intentions and behavioral responses through the measurement of the strength of the government’s role and highlights the impact of the interaction between the two actors on policy implementation. The innovations of this paper include, first, extending the previous research model of single actor in the theory of planned behavior and constructing a model of behavioral response mechanism of multiple actors to describe the whole process of energy transition policy implementation from the perspective of willingness to implement policy; and second, using structural equations to measure the respective role strengths of farmers and government and the interaction strengths between them to quantify the role strengths of actors in policy implementation, which provides a more intuitive perspective.

## 2. Model Design and Research Hypothesis

### 2.1. Model Design

In the implementation of public environmental policies, there is a universality of deviation between public behavior will and policy implementation results [20,21]. This universality lies in the different decision-making goals of subjects, resulting in different interest demands. The policy implementation of local governments is the result of policy implementation composed of positive “policy benefits” and matching financial resources under the constraints of central policy objectives and performance appraisal [22]. We use policy characteristics to describe the motivation in the implementation of government policies. Its execution power comes from the financial subsidy and assessment system of the superior government. The stronger the execution is, the more obvious the effect of energy transformation will be [23]. According to the energy ladder theory, the upgrading of energy consumption is related to income, so the choice of energy consumption considers the economy of energy [24]. However, rural household energy consumption is not only a purely economic phenomenon, but also a social psychological phenomenon [25]. The relationship between farmers’ psychological factors and behavioral intentions is also constrained by government objective factors such as institutions and policies in practice [26]. Therefore, farmers’ behavioral responses are the result of the joint action of government policy characteristics and farmers’ behavioral intentions. If there is a lack of government implementation or farmers’ intentions to participate, it will lead to policy failure.

**Figure 1:**
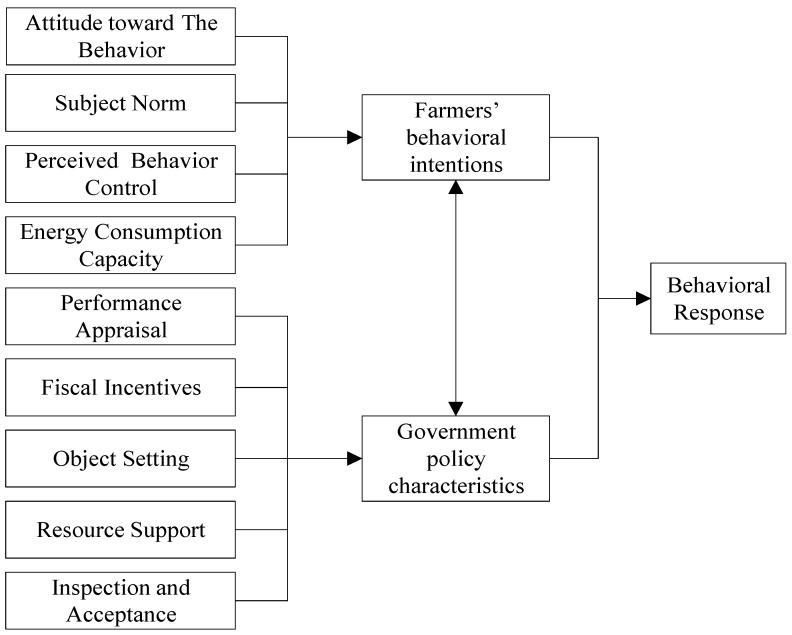
Diagram of the theoretical framework of behavioral intentions and implementation outcomes of energy transition policy.

In order to reveal this universality, this paper breaks through the existing research perspective and starts from the study of inter-subject interaction, using farmers’ behavioral willingness to reflect farmers’ spontaneous choice and government policy characteristics to reflect the strength of government’s role, and constructs a theoretical model of government and farmers’ interaction formed under different motives, as shown in Figure 1. Figure 1 contains two path clues. One is the study on the impact of policy implementation on the behavioral response of farmers in the process of passive energy transition, with the purpose of studying the mechanism of energy transition under the interaction of multiple actors (farmers’ behavioral intentions → behavioral response ← government policy characteristics). The second is the interaction between the two actors of farmers and the government, aiming at studying the interaction mode and intensity between different actors, in order to explore how to better promote the effective scheme of energy transformation (government policy characteristics ↔ farmers’ behavioral intentions). 

Specifically, farmers form their own willingness to transform according to their own behavioral attitudes, subjective norms, and consumption behavior capabilities, thus providing guidance for behavioral responses (corresponding to hypothesis H1); local governments are influenced by financial subsidies, performance assessment, and monitoring mechanisms from higher levels of government, as well as their own resources, and eventually form their own awareness of transformation, and influence the behavioral response results of farmers through the role of government (corresponding to hypothesis H2); in the process of interaction between government and farmers, the final behavioral response results are formed by both government policy characteristics and farmers’ behavioral willingness (corresponding to hypothesis H3).

### 2.2. Research Hypothesis

#### 2.2.1. Farmers’ Behavioral Intentions and Behavioral Responses

Farmers’ behavioral intentions are the behavioral motivation for farmers’ behavioral responses and are the most direct factor for the occurrence of the actual behavior. Previous studies have confirmed that intention has a direct positive effect on behavior, and the stronger the behavioral intentions, the more likely it is to perform the behavior [27,28]. In the process of energy transition, behavioral intentions are the spontaneity of farmers’ participation in energy transition, and the stronger the behavioral intentions of farmers, the more obvious the transition effect.

First, behavior attitude is the positive or negative evaluation of the individual’s behavior results. The more positive the farmers’ attitude towards environmental protection, the stronger their will to act [29,30]; Secondly, subjective norms, that is, social pressure perceived by individuals when making behavioral decisions, are usually positively related to pro-environment or energy behavior [31,32]; In addition, perceptual behavior control represents controllability [33]. Therefore, the less difficult it is for farmers to expect the energy transformation, the more confident they are in their energy knowledge, the more controllable the cost they can bear, and the stronger their intentions to participate; Finally, non-psychological factor variables are added. The energy consumption behavior ability variable refers to the potential and confidence of farmers in making energy transition decisions, namely, the receptivity, economic strength, skill level and security knowledge required to achieve decision-making. The stronger the capacity of energy consumption behavior, the more obvious the intentions of farmers to act [34]. Based on this, this paper proposes the following hypothesis:

**H1.**  *Farmers’ behavioral intentions have a significant positive effect on farmers’ behavioral responses.*

#### 2.2.2. Government Policy Characteristics and Behavioral Responses

The self-interest of local governments is, to some extent, the driving force behind the implementation behavior [35]. This paper defines the driving force of implementing policies as the characteristics of government policies. The motivation for local governments to implement public policies comes from many aspects. For example, Wang [36] believed that financial resources and policy benefits were the main reasons for local governments to actively or even over-implement the “coal to gas” policy; Feng [11] believed that in addition to the changes in financial subsidies that will affect the implementation effect of the policy of “coal to gas”, the improvement of a series of security systems such as public services is also an important aspect that affects the implementation of the policy; Wei [37] believed that the supervision and inspection work carried out by the superior government will also affect the government’s policy implementation. The stronger the supervision and inspection work, the stronger the government’s implementation motivation; Xie [38] believed that the winter bulk coal replacement project requires a lot of infrastructure construction, so the resource matching of local governments will affect the policy implementation effect.

The research shows that the government’s policy characteristics have a positive role in guiding farmers’ behavior response, and the government’s strong policy implementation can help those farmers who are willing but unable to participate to act [39]. Local governments as policy implementers, the results of policy implementation are reflected in the behavioral responses of farms. The status of farmers’ passive energy transformation highlights the role of government, so it is of great significance to study the characteristics of government policies. Based on the above literature, this paper measures the characteristics of government policies from the aspects of financial subsidies, performance appraisal, resource matching, security system and supervision mechanism, and puts forward the following hypothesis:

**H2.**  *The policy characteristics of the government have a significant positive effect on policy implementation.*

#### 2.2.3. Interaction of Farmers’ Behavioral Intentions and Government Policy Characteristics

Policy implementation is the process of a multi-agent game, with interactions between actors [22]. The implementation of public policies is a dynamic process, and the game among interested subjects has always been the basic perspective in describing the implementation of China’s policies [40]. With two different actors, the smaller the losses of both sides in the game process, the more conducive it is to policy implementation.

The government’s policy characteristics and farmers’ behavioral intentions influence each other. On the one hand, the government needs to build supporting infrastructure, vigorously promote the “coal to gas” work, so that farmers can learn more about natural gas knowledge, add energy subsidies to alleviate farmers’ pressure, strengthen after-sales service system and other work, and constantly adjust the policy implementation according to farmers’ behavioral intentions; On the other hand, the level of farmers’ behavioral intentions will also affect the implementation of government policies. The most direct influencing factors of farmers’ behavioral intentions are the conversion costs arising from the conversion of heating methods and the inertial thinking formed by the combustion of bulk coal, which cannot be accepted in a short time. This is reflected in the government’s need to fully consider the energy conversion costs of farmers. In addition, safety is also an important factor affecting farmers’ behavioral intentions. The open-air gas pipeline and fear of natural gas affect farmers’ behavioral intentions. A sound government security system is an important way to improve farmers’ behavioral intentions. To sum up, the two actors, government, and farmers, interact with each other and jointly affect the energy transformation. Based on this, this paper proposes the following assumptions:

**H3.**  *There is a significant correlation between the government’s policy characteristics and farmers’ intentions.*

## 3. Research Methods and Data Collection

### 3.1. Implementation of Policies in the Study Area

Rural energy transformation mainly includes gas coal substitution, electricity coal substitution, clean coal replacement, etc. Following the policy of “according to local conditions”, the National Energy Administration announced the list of the first batch of “pilot cities for clean winter heating in northern areas in 2017” [41]. As a city close to Beijing, the capital of China, Hebei is one of the most important cities in the comprehensive management of air pollution, so Shijiazhuang, the capital of Hebei Province, was chosen as the first pilot city to participate in the energy transition, and the transition method adopted is mainly “coal to gas”. Among them, Gaocheng District and Luancheng District are close to the main urban area and have a better level of economic development compared to other rural areas, so they have become demonstration areas for the Shijiazhuang Municipal Government to implement energy transition. Therefore, Gaocheng District and Luancheng District, as typical study areas of “coal to gas” policy implementation in Hebei, are highly representative and can provide reference values for energy transition in other regions in northern China. 

In this special project for air pollution control, a total of sixteen medium pressure pipe networks were newly built in Gaocheng District, covering 75 natural villages, and 63,135 households were actually installed. Luancheng District’s “coal to gas” special project involves the jurisdiction of 133 natural villages, and a total of 56,860 households. To reduce the gas consumption cost of farmers, a unified subsidy policy has been introduced, including: 

(1) Subsidizing purchases and installation of facilities. Each household is subsidized 1000 CNY for purchasing and installing heating facilities. Households are required to purchase wall-mounted gas heaters designated by the city government and have to self-pay for expenses that exceed the subsidy. 

(2) Operating subsidies. The operating subsidy policy is implemented for a period of three years. From 2017 to 2019, residents were subsidized 1.4 yuan for every 1 m^3^ of gas used, for a maximum of 1680 yuan. From 2019 onwards, the subsidies declined annually, that is, 50% less in the first year, 75% less in the second year, and no subsidy in the third year. 

### 3.2. Scale Design

A questionnaire was developed in this study based on the theoretical analysis results and on-site conditions. The behavioral attitudes dimension was revised according to the study by Kaiser (1999) [42]; the subjective norms dimension was revised according to the study by Im (2011) [43]; the perceived behavioral control dimension was revised according to the study by Zhang (2020) [39]; the behavioral capabilities dimension was revised according to the study by Wu (2016) [34]; and the government’s behavioral intentions dimension was revised according to the study by Wang et al. [22,23,36]. The behavioral responses of the farmers were expressed based on the actual policy implementation and government inspection and acceptance status. A value of 1 was assigned to a “yes” response, and 0 to a “no” response. The latent variables were measured on a five-point Likert scale, in which the values of 1 to 5 corresponded to a response of “completely disagree,” “disagree,” “somewhat agree,” “agree,” and “completely agree.” The questionnaire is presented in Table 1 and Table 2.

### 3.3. Data Collection

The data comes from the questionnaire survey of farmers in Gaocheng District and Luancheng District of Shijiazhuang who participated in the “coal to gas” project. Twenty-three villages from the six towns in Gaocheng and Sixteen villages from the five towns in Luancheng were randomly selected. The study group conducted semi-structured interviews with participants and administered the questionnaire to each of the sampled farmers. The meanings of the questionnaire items were explained to the farmers on the spot. A total of five hundred questionnaires were administered and 470 were recovered (an effective response rate of 94%), of which 420 were from farmers and 50 were from government workers.

The questionnaire contents covered the farmers’ basic family background, age, education level, housing condition, and income. The farmers’ willingness to participate in the coal-to-gas transition included the changes in their cost of heating, the percentage of expenditures spent on energy, knowledge, satisfaction with the subsidies. The local government’s willingness to implement the coal-to-gas policy included the status of policy implementation, the performance evaluation methods, and the status of resource allocation.

Lastly, the data of farmers and government workers were matched. Even though both are different behavioral actors, they have a common key variable (behavioral response) and the policy features in the same area can be merged horizontally due to their heterogeneity [44]. On this basis, the data of farmers and government workers were matched horizontally by area to ensure that all farmers were under the jurisdiction of the government workers in the respective study areas [45]. The details are specified in Table 3.

### 3.4. Validation of Model Validity

Model reliability and validity were performed with SPSS22.0 software. Reliability was expressed through the Cronbach’s α, which, as recommended by Hair et al. (1978) [46], is deemed acceptable when having a value of 0.7 or above and must be rejected if smaller than 0.35. The overall Cronbach’s α in this study was 0.940 and all the individual Cronbach’s α of the latent variables exceeded 0.6, which suggests that all the latent variables and the internal consistency of the model have excellent reliability. To ensure the overall rationality of the questionnaire, exploratory factor analysis was performed on the text, and the resultant Kaiser–Meyer–Olkin (KMO) statistic was 0.962, the Bartlett’s test of sphericity result was 5671.93, and the level of significance was 0.000. These figures demonstrate the robust validity of the questionnaire. The results are presented in Table 4. 

### 3.5. Model Estimation Results

To check whether SEM is suitable for analyzing the willingness of farmers and the government in energy transition, the overall fit of the model must be assessed. The goodness-of-fit test results obtained through AMOS23.0 software are shown in Table 5. As all the indicators met the measurement criteria, the fit of the model was considered decent.

**Figure 2:**
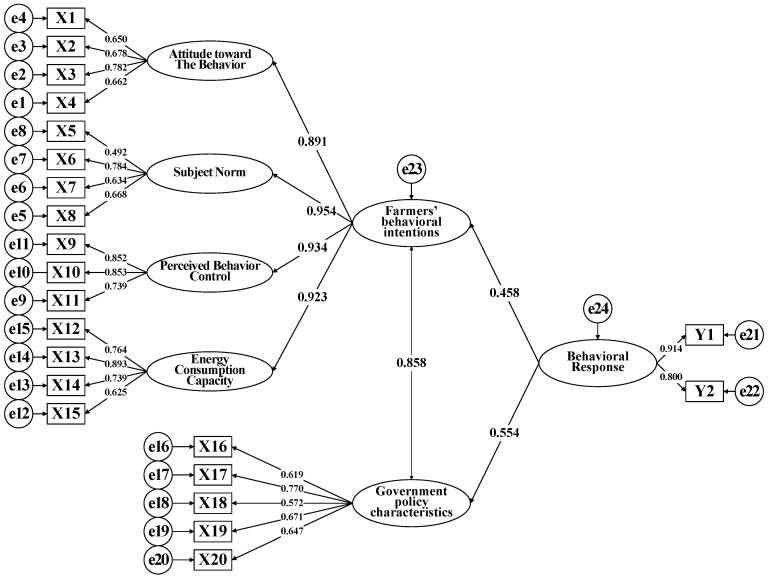
Structural equation model and normalized path coefficient diagram.

The SEM analysis results are shown in Table 6, the path coefficients of the model are presented in Figure 2. From Figure 2, the willingness of farmers and the government had a positive effect on the farmers’ behavioral response, with the standardized path coefficients being 0.458 at a 1% level of significance and 0.554 at a 1% level of significance. Thus, H1 and H2 are supported. The correlation coefficient between farmers’ behavioral intentions and government policy characteristics was 0.858, and hypothesis H3 passed the test.

## 4. Results and Analysis

### 4.1. Analysis of the Farmers’ Behavioral Intentions

#### 4.1.1. Analysis of the Strength of the Farmers’ Behavioral Intentions

**Figure 3:**
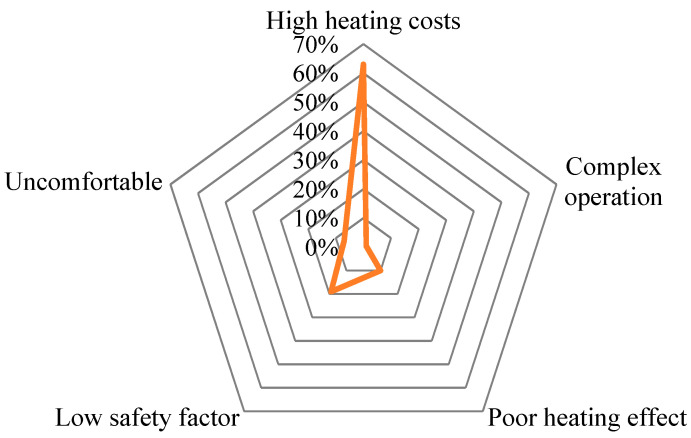
Survey on farmers’ dissatisfaction after the implementation of “coal to gas”.

As shown in Figure 3, the standardized path coefficient of the effect of the farmers’ behavioral intentions on their behavioral response was 0.458 at a 1% level of significance. This finding indicates that the strength of the farmers’ behavioral intentions in their behavioral response toward the coal-to-gas policy was 0.458. The farmers’ behavioral intentions represent their autonomy-based logic of behavior. The results suggest that there is a positive and significant correlation between both variables. Based on the on-site interview data, the reasons behind the farmers’ dissatisfaction with using gas for heating are the high cost of heating (64%), the low safety factor (19%), and the low heating efficiency (10%) (see Figure 3). This shows that the high cost of heating is the main concern among the farmers, and the outdoor gas pipelines are also a safety concern. The poor heating efficiency is a result of the structure, floor area, and compactness of the farmers’ homes, which suggests that livelihood issues also reduced their experiential value.

#### 4.1.2. Analysis of Factors Influencing the Farmers’ Behavioral Intentions

(1) Effect of behavioral attitudes on farmers’ behavioral intentions. As shown in Figure 2, among the factors influencing behavioral attitude, heating cost issues had the highest load factor at 0.782. This signifies that the cost of energy consumption affects behavioral attitudes. Furthermore, due to the farmers’ general lack of awareness toward environmental protection, they have much room to grow regarding their responsibility in air pollution governance. As this is strongly associated with their education level, ideological consciousness, and policy promotion, strengthening their environmental protection awareness can enhance energy transition to a certain extent.

(2) Effect of subjective norms on farmers’ behavioral intentions. The load factor of family mindset was the highest (0.784) among all the factors of subjective norms, followed by information spreading among farmers (0.634). These findings are in line with traditional Chinese customs and habits, thus highlighting the need to redress the farmers’ mindsets. In addition, the widespread bandwagon-jumping effect in Chinese societies in which neighboring farmers follow the actions of one another also changes the farmers’ behavioral intentions.

(3) Effect of perceived behavioral control on farmers’ behavioral intentions. Among the three factors influencing perceived behavioral control, cost-related beliefs had the highest load factor with 0.853, followed by perceived difficulty with 0.852. One of the sources of the farmers’ risk-resisting capability is their own nature while the source is government support. Therefore, two ways to enhance the farmers’ behavioral intentions are simultaneously considering the roles of both behavioral actors (farmers and the government) and assisting farmers to undergo transition on their own initiative. These approaches can expedite the enhancement of the energy consumption structure in rural households.

(4) Effect of consumption capability on farmers’ behavioral intentions. Among the factors influencing the farmers’ consumption capability, the farmers’ financial status had the highest load factor with 0.893, suggesting that their financial status is the primary factor that dictates their willingness to undergo energy transition. Therefore, the government should instruct gas companies to strengthen their demonstrations of operational procedures and safety training to enhance the farmers’ behavioral capabilities.

### 4.2. Analysis of the Government Policy Characteristics

#### 4.2.1. Analysis of the Strength of the Government Policy Characteristics

Figure 2 shows that the standardized path coefficient of the effect of the government’s policy characteristics on the farmers’ behavioral response was 0.554. Hence, there is a positive and significant correlation between both variables. The government’s effect on the farmers’ behavioral response was simultaneously facilitative and restrictive [39], which demonstrates the complexity of the farmers’ decision-making process.

First, the government plays the important role of a mediator in the process of energy transition in rural villages. As the implementer of the coal-to-gas policy, the government on the one hand must consider the farmers’ thoughts and opinions, and on the other hand, must cooperate closely with gas companies. During our surveys of the low-ranking government workers, when the topic was on the farmers’ willingness to comply with the policy, the government workers expressed that most of the farmers were unwilling to cooperate and adamantly stuck to their traditional mindset of coal as a superior fuel for heating. The statistics on the farmers’ willingness to comply with the policy are presented in Table 7. Those with a low willingness accounted for 21.06% of the sample. The gas companies reported episodes of noncooperation and confrontation among the farmers toward the gas company employees when they were installing the facilities. This highlights the need for government officials to negotiate with the farmers and fulfill their duties as a responsible government.

Second, the characteristics of government policies lead to the deviation between farmers’ behavioral intentions and behavioral response. According to the theory of planned behavior, willingness is behavior. However, as shown in Figure 2, there is a deviation between farmers’ behavioral willingness and behavioral response, and the reason for this deviation is the emergence of the role of the government. Moreover, the standardized path coefficient between the government and farmers is 0.858, which passes the significance test at the 1% level. It shows that the two actors influence each other. The government adjusts the policy implementation plan according to the behavior intentions of farmers, and farmers influence the behavior intentions of farmers according to the characteristics of government policies. There is a strong correlation between the two actors. This further indicates that the difference between farmers’ behavioral intentions and behavioral response is caused by the characteristics of government policies.

#### 4.2.2. Analysis of the Factors Influencing the Government Policy Characteristics

Figure 2 shows that the two leading factors influencing the government’s policy characteristics are financial stimulus and resource configuration, with a load factor of 0.770 and 0.671, respectively. The details are as follows: 

(1) Financial stimulus. Against the backdrop of political centralization and fiscal decentralization in China, local governments compete against one another with the hopes of acquiring the central government-allocated air pollution governance funds [47]. The central government subsidizes city governments based on the city level, with municipalities receiving one billion yuan annually, provincial capitals receiving 700 million yuan, and prefectural cities receiving 500 million yuan. The central government’s incentives for replacing coal with gas are the direct driving force behind the local governments’ policy implementation and significantly enhance their willingness to implement the coal-to-gas policy [36]. In the interviews, the low-ranking government workers disclosed that because the subsidy policy changes every three years, they were unsure about the content of upcoming policies and preferred to implement the policy while the subsidies are still available. This situation explains why gas replacements surpassed targets in 2017 and resulted in widespread gas-powered heating equipment shortages [23].

(2) Resource configurations. Resource configurations include local financial allotments and the gas supply status. The interview data revealed that the farmers’ energy subsidies were distributed across the province, city, and prefecture levels, and the local governments must defray some of the costs. When asked about their opinions toward the policy, most of the farmers complained about the untimely nature of the subsidies. The gas companies also reported that the subsequent maintenance costs in the villages were too high and could not be covered by the government’s one-time subsidies, thus resulting in “backfires.” In addition, the local governments must also bear the cost of hiring safety taskforces, which requires sufficient capital. The interviewed farmers also claimed that gas supply interruptions were rampant in 2017 and 2018, particularly for the low end-point air pressure and the unstable gas supply. According to the gas company employees, these situations were directly caused by the outperformance of the coal-to-gas transition, in addition to the increased heating demands during the winter months, which exceeded the initial plans. Therefore, the capability of local governments to configure their resources strongly affect the outcomes of the energy transition.

### 4.3. Interaction Analysis of Two Actors

#### 4.3.1. Analysis of the Influence of Government Actions on Farmers’ Behavioral Intentions

As shown in Figure 2, the standardized correlation coefficient between farmers’ behavioral intentions and government policy characteristics is 0.858, indicating a high correlation. This indicates that farmers and the government, as two important actors in the “coal-to-gas” policy, complement each other and work together to complete the energy transition in rural areas.

(1) Government propaganda work to guide farmers’ behavioral intentions

The publicity and guidance of the government can improve the willingness of farmers to transform [11]. Local government officials said that to do a good job of publicity work, in addition to handing out leaflets and broadcasting, but also face to face with farmers to answer questions, interpretation of national policies; Set up the information desk, carry out the field knowledge award question and answer, and release small gifts. The municipal government cooperated with the Education Bureau to conduct gas safety education activities for more than 30,000 middle and primary school students, and asked parents to take their children to complete the investigation of hidden dangers of natural gas detection and hand in their signatures. During the peak heating season, the united area television stations broadcast safety videos and other activities during the prime time. These publicity work promoted the process of “coal to gas” and improved the farmers’ behavioral intentions.

(2) Government guarantee system to increase farmers’ behavioral intentions

The government guarantee system includes after-sales service guarantee and operation subsidy implementation guarantee. Government staff said that to solve the maintenance delay, to provide “energy + equipment + operation and maintenance + training” and other comprehensive service system, to build a perfect security system. First, establish after-sales service WeChat group, and set up specialized maintenance personnel to explain; Second, the gas company added customer service personnel to guide farmers to solve problems by themselves; Third, increase the village safety staff training work, reduce short failure time. These government services and guarantee work have improved farmers’ behavioral intentions.

(3) the intensity of government supervision affects the farmers’ behavioral intentions

According to the interviews with government staff, the government’s supervision of farmers is mainly reflected in the control of coal burning, including recycling coal boilers, restricting heating methods, eliminating the return of coal for heating, and regulating the heating behavior of farmers through administrative means. Set up the supervision team, the joint village committee to take the form of field inspection, increase supervision and inspection. In addition, in the construction of the “coal to gas” project, the construction unit shall submit the project progress before the 5th of each month as required to ensure the process of energy transformation. These powerful regulatory measures of the government have restrained the behavior of farmers, and finally improved the behavior intentions of farmers.

#### 4.3.2. Analysis of the Impact of Farmers’ Intentions on Government Behavior

(1) Farmers’ demands influence government policy characteristics 

On the one hand, farmers are worried about the timeliness and sustainability of the subsidy policy. According to the research, the heating cost after the change from coal to gas is about three times that of coal [48]. In field interviews, many farmers said they could not afford to pay without government subsidies. After the expiration of the three-year subsidy policy, the municipal government set up a downslope mechanism to gradually ease the pressure on heating costs. Another interest mainly reflects the aspect of security worries, appeal to the government to continuously perfect the safety of farmers. For example, adding to the inspection of the service work, providing farmers with a security inspection services, and consulting services, through-the-door inspections, screening of various hidden dangers in time, and gas to protect the safety of rural users. These government actions are adjusted according to the interests of farmers to protect the interests of farmers.

(2) the attitude of farmers affects the implementation ability of government

During the interviews, it was determined that in the early stage of the implementation of the policy of “replacing coal with gas”, farmers had an aversion to energy transformation due to their habitual thinking in terms of burning coal for heating. During the project construction, there were often conflicts with the construction units, and government personnel were needed to mediate. In order to accelerate the pace of energy transformation, the government starts with non-farmers, gives play to the leading role of the masses, and enables farmers to experience the advantages of clean and sanitary natural gas, convenient and quick cooking, and simple operation, so as to correctly guide farmers’ environmental values and gradually change their attitude from negative to positive. Government officials stated that in the second phase of the project, farmers’ behavior attitude was increasingly positive, the number of mediations decreased significantly, and the government’s enforcement power became stronger and stronger. It fully reflects the effects of farmers’ attitude on the government’s executive ability.

(3) Farmers’ satisfaction affects the effect of policy implementation 

Farmers’ satisfaction is in the user experience. In the research process, many farmers indicated that the payment method is not convenient, due to the winter heating season payment being more frequent, and the service station payment queue being too long. According to the local business network staff learned that in Lianzhou Town, of the current 24 villages, only 5 villages can pay online, and there is a problem with inconvenient gas purchase. In this regard, the government staff said that in order to facilitate rural users to purchase gas, business outlets can carry out a variety of special services as soon as possible to broaden the online payment area, and strive to become all the Internet able, both to simplify the workload of the gas company staff meter reading, but also to increase the satisfaction of farmers.

## 5. Discussion

In the process of energy transition policy implementation, the government acts as the executor of the policy and farmers act as the recipient of the policy, so the energy transition policy implementation is accomplished under the joint role of two important actors, the government, and farmers. The analysis of the behavioral willingness and interaction strength of the government and farmers can provide the following information:

(1) it reveals the reasons for the deviations between farmers’ behavioral intentions and behavioral responses. The theory of planned behavior argues that willingness is behavior. In this paper, by constructing a theoretical model of energy transition policy implementation with the interaction between government and farmers and measuring it using structural equations, the strengths of the effects of farmers’ behavioral intentions and government policy characteristics on farmers’ behavioral responses are 0.458 and 0.554, respectively, highlighting the influence of the government’s role on the change of farmers’ behavioral responses and further revealing that the reasons for the deviations between farmers’ behavioral intentions and behavioral responses are due to the role of government.

(2) the interactions between actors in energy transition policy implementation were quantified. In policy implementation, the interaction between the government and farmers jointly affects the effectiveness of energy transition. This paper calculated the interaction intensity between the government and farmers as 0.858. The higher intensity reflects that the interaction behavior between the government and farmers has a significant impact on the policy implementation results, which verifies the importance of studying the interaction between the government and farmers actors and provides a new perspective on how to improve the energy transition policy implementation effectiveness.

However, there are some limitations in the research process. In terms of research methods, there are two behavioral subjects in the theoretical model built based on the theory of planned behavior, the influence of behavioral intention on the results of behavior is statistically subjective, and individual social psychological deviation may also form a certain degree of deviation on the results of policy implementation. In terms of data acquisition, because the data come from farmers’ subjective judgment and farmers filled out the questionnaire using their own memory, there are certain errors, so it is not as accurate as the data obtained from the longitudinal study, and there are certain shortcomings. To ensure the accuracy of the data as much as possible, this paper treated objective and subjective indicators differently. The indicators of occupation, income, and education level were filled in truthfully according to the actual situation of farmers. For the subjective indicators such as willingness to transition to energy and satisfaction with energy subsidies, and because Chinese rural areas are relational network societies with each family knowing each other in detail, in addition to the description of the farmers’ own situation, the evaluation results of others should be added to correct them, so as to avoid farmers’ falsification and ensure the true validity of the research data.

## 6. Conclusions and Policy Recommendations

The existing literature analyzes government activism in energy transition more from the perspective of government subsidies, ignoring the interactive role between government and farmers, and failing to dissect the prevalence of such deviations. To this end, this paper gives a new perspective on inter-subjective interactions, constructs a model of government–farmer interactions in rural passive energy transition, systematically analyzes the influencing factors affecting farmers’ behavioral responses from both government and farmers, and uses structural equations to measure the strength of the inter-subjective effects. The model is validated by taking the “coal-to-gas” policy as an example, and the following conclusions are obtained:

(1) the standardized path coefficients of farmers’ behavioral intentions and government policy characteristics on farmers’ behavioral responses are 0.458 and 0.554, respectively, and the intensity of government role is higher than that of farmers’ role, which highlights the influence of government role on the change of farmers’ behavioral responses and better explains the prevalence of deviations between farmers’ behavioral intentions and behavioral responses.

(2) the correlation coefficient between government policy characteristics and farmers’ behavioral intentions is 0.858, and the validation result shows a significant correlation, emphasizing the mode of action and the intensity of action between the two. The higher correlation coefficient indicates that the interactive behavior between the government and farmers has a significant effect on policy implementation results.

Through the fitness test of the model, the theoretical model proposed in this paper is feasible. Based on this, to improve the effectiveness of energy transition, the following policy recommendations can be derived from the consideration of the interaction between government and farmers.

First, improve the willingness of farmers to transform themselves and reduce the intensity of the government’s role. For how to improve farmers’ willingness to act, on the one hand, increase the publicity of rural energy transformation, improve farmers’ awareness of environmental protection, so as to change behavioral attitudes; on the other hand, make full use of farmers’ psychology of emulation, do a good job in the ideological work of household heads, so that farmers can realize the transformation from passive transformation to active transformation, reduce the government’s mandatory implementation, and strengthen the construction of subjective norms; finally, strengthen the work of operation demonstration, safety training, etc. ensure heating safety, improve farmers’ behavioral ability, and finally change farmers’ satisfaction.

Second, smooth the channels for farmers’ demands and strengthen the interaction between farmers and the government. The current government-led energy transition has weakened the role of farmers and ignored their participation and supervision rights. For better communication between the government and farmers, the government should pay more attention to farmers’ interest demands and adjust government policy behavior according to their demands. For example, if farmers are most worried about energy subsidies, the government should give more consideration to the timeliness and sustainability of the subsidy policy, cultivate the habit of natural gas users, and relieve the pressure of rising heating costs. Therefore, the government should establish a channel for farmers to express their interests, and form a government-led, all-participant air pollution control pattern.

## Figures and Tables

**Table 1:** Design of the scale of Farmers’ behavioral intentions.

Latent Variable	Number	Measurement Topics
Attitude toward The Behavior	X1	Bulk coal combustion is the main cause of air pollution in winter
	X2	“Coal to gas” has improved environmental quality, and we should actively participate in it.
	X3	I can accept the cost of “coal to gas”.
	X4	The government promotes the policy, as a qualified citizen should cooperate
Subject Norm	X5	The surrounding neighbors have implemented “coal to gas”, We should also implement
	X6	The use of natural gas has facilitated daily life and the family supports the installation
	X7	Hearing that many places have already implemented “coal to gas”, we feel that it is okay
	X8	The village committee actively promotes the advantages of “coal to gas”, which is acceptable.
Perceived Behavior Control	X9	I do not think it’s too difficult to participate in the “coal-to-gas” conversion.
	X10	The increase in heating cost is affordable according to your situation
	X11	Government subsidies give strength to overcome difficulties
Energy Consumption Capacity	X12	Energy consumption as a proportion of total household consumption is acceptable
	X13	My income can pay for the increase in “coal to gas” costs
	X14	I have a good idea of how to use natural gas
	X15	The safety hazards that exist can already be avoided
Behavioral Response	Y1	Whether the completion of the “coal to gas” project
	Y2	Superior government inspection and acceptance

**Table 2:** Design of government policy characteristics.

Latent Variable	Number	Measurement Variable	Measurement Topics
Government policy characteristics	X16	Performance Appraisal	The government has built a strict pre, during and post assessment system
	X17	Financial Stimulus	Superior financial allocation and rewards
	X18	Object Setting	The government has set a detailed target plan for this “coal to gas”
	X19	Resource Configurations	Energy consumption subsidies in place and the availability of gas
	X20	Inspection and Acceptance	The implementation of the “coal to gas” policy, the situation of burning coal and other supervision and inspection

**Table 3:** Descriptive analysis of the basic characteristics of the sample.

Index	Classification	Quantity	Proportion
Age	Under 30	24	5.11%
	30–60	350	74.47%
	Over 60	96	12.34%
Region	Luancheng District	200	42.55%
	Gaocheng District	270	57.45%
Farmers’ income Sources	Non-agricultural income ≤ 30%	116	24.68%
	30% ≤ Non-agricultural income ≤ 90%	260	55.32%
	Non-agricultural income ≥ 90%	94	20.00%
Type of house structure	Brick-concrete	306	65.11%
	Brick and wood	73	15.53%
	Reinforced concrete	91	19.36%
Monthly income level	Under CNY 3000	121	25.74%
	CNY 3000–5000	296	62.98%
	Over CNY 5000	53	11.28%

**Table 4:** Results of factor analysis.

Variable	Cronbach’s α	KMO
Attitude toward The Behavior	0.751	0.751
Subject Norm	0.639	0.689
Perceived Behavior Control	0.825	0.693
Energy consumption capacity	0.822	0.771
Government policy characteristics	0.707	0.763
Behavioral response	0.845	0.500

**Table 5:** Model goodness-of-fit tests.

Statistical Test Volume	Actual Fitted Value	Metrics	Fitting Evaluation
Absolute fit index			
GFI	0.880	>0.8	Satisfactory
AGFI	0.852	>0.8	Satisfactory
Relative fit index			
CFI	0.914	>0.9	Satisfactory
TLI	0.903	>0.9	Satisfactory
IFI	0.914	>0.9	Satisfactory
Parsimonious fit index			
RMSEA	0.071	<0.08	Satisfactory
SRMR	0.054	<0.08	Satisfactory

**Table 6:** Results of model hypothesis testing.

Hypothesis	Standardized Path Coefficient	S.E.	C.R.	P
Behavioral response ← Farmers’ behavioral intentions	0.458	0.135	5.542	***
Behavioral response ← Government policy characteristics	0.554	0.146	6.336	***
Farmers’ behavioral intentions ↔ Government policy characteristics	0.858	0.036	8.975	***

Note: *** indicates passing significance test at 1% level.

**Table 7:** The willingness of farmers to implement the policy of “coal to gas”.

Willingness to Perform	Proportion
Very willing	12.55%
Willingness	38.72%
General	27.66%
Unwillingness	12.55%
Very unwilling	8.51%

## Data Availability

The data presented in this study are available on request from the corresponding author. The data are not publicly available due to the data involve sensitive information such as the income and perceived status of the farmers.
